# Discussions in the Mississippi Valley Dental Association

**Published:** 1873-04

**Authors:** 


### PROCEEDINGS OF SOCIETIES.

### Discussions in the Mississippi Valley Dental Association.

At the twenty-ninth annual meeting of this association the discussion of subjects reported for that purpose by the Executive Committee, consisting of Drs. H. A. Smith, J. Knapp, and W. P. Horton, was duly had, and the subjects disposed of in so far as the time permitted. The following subject was first taken up:

"Artificial Dentistry; should it be practiced as a specialty? what may be done to raise the standard of operations in this department?"

Dr. Frank A. Hunter opened the subject by reading a paper.

Dr. H. R. Smith called attention to the fact that the time was when a great deal of attention was occupied by the examination of specimen metallic plates; but where are they now? The trouble was, that dentists were discriminating against mechanical dentistry; or, if it received their attention, they kept their knowledge to themselves, and did not impart any of it in the societies and associations. For his part he believed in telling all he knew, so as to benefit the profession to the extent of his ability.

Dr. J. Taft thought it was not because mechanical dentistry was less thought of than formerly that it did not receive attention, but rather because a higher appreciation was set on the natural teeth, and greater effort was made in the direction of saving them; in proportion as dentists succeed in saving those they paid less attention in the former branch. He could not, however, overlook that as a dentist was called upon to practice both branches, the operative and the mechanical he should be prepared to do his best in both. It was not given to all to be perfect in both; some excelled in one branch only. There was a good deal in native ability. A man who could concentrate his power upon one thing, who could command every faculty and acquirement, and bring them to bear upon the performance of whatever he undertook, was sure to succeed. He believed that it was better for the majority to confine themselves to one or the other branch, because the field is wide enough to draw out the ability, and to occupy the time of the dentist. The mechanical branch had become very much demoralized in the last ten years. What is its object? Simply to supply the loss of the teeth, to restore the contour of the face, to restore the function of the teeth. The great majority of rubber sets failed to do this in every particular. So little care was taken in making them, that the workman might just as well do the work without ever looking into the patient's mouth. No attention was giAen the several oonditions required. For example, the miserable manner in which enunciation was secured. Indeed, so poor was this, that one could tell by the mumbling at midnight that the person wore a bad artificial set. Yet teeth can be made so as to restore the speech. But what is to be done to effect this desirable result? In his opinion it consisted in going down to the bottom, and requiring of dentists a knowledge of the fundamental principles. Where was the man with a grain of sense, who would call a man an artist, who painted a portrait of a loved one with the nose turned one way, and the mouth another, much less take it for a likeness? Oh, but some might take it because it was cheap! Very well, that might do with some; but it is not the right way in art, nor would it be in dentistry.

Dr. James Taylor said he had not given much attention of late years to mechanical dentistry, and had never himself made a hard rubber case. He considered the mechanical dentist an artist in the strict sense, and that it was impossible to make good work with blocks, except the dentist himself made the blocks to suit. The question was how they were to meet the difficulty? The public taste is vitiated. He believed there was higher art in the mechanical branch, yet how could the public be taught to believe so?

Many think it makes very little difference whether they mumble their words or not, or whether articulation is destroyed. He did not believe a good mechanical dentist could be a good operative dentist, because he did not possess the artistic taste.

It was his opinion that something must be done to redeem the mechanical branch, or it would eventually run into the hands of quacks and steam dental shops.

Much of the work now done is put in for $20 or less, even for those who can afford to pay for having a good set. In conclusion he regarded the whole matter as in the hands of the dentist to direct the minds of his patients to a higher and better standard. He should say to them what the work should be, and firmly refuse to do a meaner job, or put in an inferior article. He wanted it understood that he would not warrant rubber work, and believed if dentists combined on that principle, the difficulty now existing would be remedied.

Dr. Osmond spoke in favor of a combination of gold and vulcanite; but the chair reminded him that those materials were not under discussion.

Dr. Harroun took the negative of the subject. He did not think it could be practiced as a specialty. He could not see how a man could call himself a dentist without working, or being able to work at both branches equally well; as far as he was concerned, to work at both was a necessity, and he felt refreshed and renewed by changing off. He regarded the two as going together, just like medicine and surgery.

Dr. Eames took issue. He thought surgery and medicine were sufficiently distinct, at any rate not to be cited in the present case. He could not see how the mechanical branch could be presented as a specialty. Much more was required from students now than formerly, therefore they were better qualified for making a specialty of either branch. Surgery and medicine must be separate, just as much as surgery and making wooden legs; and he believed too, that eventually the branches would be separately practiced, and that dentistry would be advanced by it.

Dr. H. A. Smith held that it was the prime duty of the dentist to preserve the natural teeth. He thought the gentlemen forgot the tendency to cheap work. He believed the two branches should be separate; cheap dentistry had brought reproach upon the profession. There was no brains in it. Anybody could do it, or learn to do it in a short time, and be able to compete with the best cheap dentists in the market. An educated dentist don't care about it, for there is no interest in it for him. He thought therefore that they should recognize the signs of the times—division of labor—and give their time to the higher duties of the profession.

Dr. Horton believed that every dentist who is educated to practice on the face should understand mechanical dentistry. Sculptors only prepared the model, and then called in an assistant to chisel it in stone or marble. Every man who is a dentist should be able to model the work, and then call a skilled assistant to his aid, if he chooses, to work out the design. The Paris barber directs how the hair shall be dressed; his assistants do it. He employed two skilled assistants under this view, and could not get along without them.

The artistic idea is utterly disregarded in the steam dental establishments; no attention whatever is paid to the suitableness of dentures to the face and voice. It was for such reasons then that he believed the branches should not be separated. There should be a thorough education for both, whether the dentist works in both or not.

Dr. Eames differed with this view. He did not think the artistic aided the dental surgeon at all; he believed the mechanical dentist should know how to do his work well.

Dr. Taylor repeated his opinion in favor of a division of the two branches, but was not prepared to say that the colleges should refuse to teach the one branch. He would not be willing to send a patient to a man who devotes his time exclusively to one branch, especially the artificial. It was his interest to pull teeth, in order to put in artificial sets, and this was really the only objection the speaker had to a division of the branches at this time.

Dr. McKellops was decidedly in favor of the separation. He thought the sooner it came the better. He did not regard the mechanical dentist as a maker of teeth merely. He must bring the teeth up to their natural position for utility and beauty. The mechanical dentist must know what is required in each particular case; he must be an artist, a man of brains and skill. If an operator can do all this too, well and good; but it seemed more likely to him that he would get things muddled up, and life was too short to be spent by one man in the useless effort to bring perfection out of both branches.

Dr. Frank A. Hunter would like to hear from the rubber workers present.

Dr. Jay said he worked in it altogether, and found it hard to get people to have anything better; whenever he proposed it, he found that they argued that as long as they could get a good enough set for twenty dollars, what was the use of paying more for any other material? He thought that people would have rubber work just as long as they can get it, and the thing would not be controlled.

Dr. Knapp thought among the causes operating against first-class mechanical dentistry was the greater attention paid to saving the natural teeth. His practice convinced him of this. He had whole families who would have nothing but gold fillings, and they would have their teeth regularly attended to. He conceded that there was little demand for good work, and it remained with dentists themselves to educate the people up to the better standard; but the trouble was it would not put money in the pocket. The only way however was to change the current of public opinion on artificial dentures.

Dr. Hall thought the public sentiment here battled against, was created by dentists themselves.

Dr. Leman had no doubt that gentlemen talked one way in public, and acted another in their offices.

He found rubber the only thing suitable for a case of cleft palate. He had no trouble with it in his practice, and was not prepared to dispense with it.

Dr. McKellops speaking to the second part of the question said, that the only way to elevate the profession was to have only educated men in it, men skilled and educated. Next, to do no cheap work, and by and by the people would begin to compare the results of the two classes of work, namely the poor and cheap work, and the artistic and best, and then fall naturally in with the best, and would have no other.

Discussion closed on this subject.

